# Interleukin 21 Receptor Affects Adipogenesis of Human Adipose-Derived Stem/Stromal Cells

**DOI:** 10.1155/2022/4930932

**Published:** 2022-01-10

**Authors:** Bruna Cristina Falavinha, María Julia Barisón, Carmen Lúcia Kuniyoshi Rebelatto, Bruna Hilzendeger Marcon, Alessandra de Melo Aguiar, Evelin Brandão da Silva, Marco Augusto Stimamiglio, Patrícia Shigunov

**Affiliations:** ^1^Laboratory for Basic Biology of Stem Cells (LABCET), Instituto Carlos Chagas-FIOCRUZ-PR, Curitiba, Paraná 81830-010, Brazil; ^2^Core for Cell Technology, School of Medicine-Pontifícia Universidade Católica do Paraná, Curitiba, PR, Brazil

## Abstract

Dysfunctions in adipose tissue cells are responsible for several obesity-related metabolic diseases. Understanding the process of adipocyte formation is thus fundamental for understanding these diseases. The adipocyte differentiation of adipose-derived stem/stromal cells (ADSCs) showed a reduction in the mRNA level of the interleukin 21 receptor (IL21R) during this process. Although the receptor has been associated with metabolic diseases, few studies have examined its function in stem cells. In this study, we used confocal immunofluorescence assays to determine that IL21R colocalizes with mitochondrial protein ATP5B, ALDH4A1, and the nucleus of human ADSCs. We demonstrated that silencing and overexpression of IL21R did not affect the cell proliferation and mitochondrial activity of ADSCs. However, IL21R silencing did reduce ADSC adipogenic capacity. Further studies are needed to understand the mechanism involved between IL21R and the adipogenic differentiation process.

## 1. Introduction

Adipose-derived stem/stromal cells (ADSCs) are considered ideal for application in regenerative therapies for several reasons, including the ease and efficiency with which they can be extracted, handled, and expanded [1]. In addition, they are a study model for understanding the mechanisms of cell differentiation. This process is induced by specific physiological conditions that influence the stem cell to perform characteristic functions and integrate specialized tissues [2]. Adipocytes are the mature cells that make up most of the adipose tissue and are formed by adipogenesis, a complex and fine-tuned process (reviewed by [3]). Adipogenesis has been extensively studied by using preadipocyte models *in vitro* to better understand their characteristics in adipose tissue health (reviewed by [4]).

Different studies have investigated the cell differentiation process by using large-scale RNA sequencing throughout the adipogenesis of human ADSCs [5–9]. We previously demonstrated that the amount of interleukin 21 receptor (IL21R) mRNA associated with polysomes decreases during early adipogenic differentiation (logFC = −1.723, FDR = 8.22*E* − 04) [5]. Another study found that the amount of IL21R mRNA decreased in the polysomal fraction of ADSCs with the activation of the Hedgehog signaling pathway and increased when the latter was blocked [10]. IL21R is a transmembrane glycoprotein with a molecular mass of approximately 60 kDa, which is part of the group of type I interleukin receptors. Its association with a common gamma chain (CD132) forms a heterodimeric receptor complex [11–14]. Interleukin 21 interacts with IL21R (its receptor) and transduces the signal into cells through proteins JAK1, JAK3, STAT1, and STAT3, which are primary participators in the JAK-STAT signaling pathway [15, 16].

IL21R is expressed in immune cells such as B lymphocyte cells, T lymphocytes, natural killers (NK), macrophages, and dendritic cells [11]. In vitro assays suggest that IL21 plays a role in the proliferation and maturation of NK cell populations from bone marrow, the proliferation of mature B-cell populations costimulated with anti-CD40, and the proliferation of T cells costimulated with anti-CD3 [11]. IL21 has also been found in several tumor cell lines, intestinal epithelium cells affected by inflammatory diseases, and gastric epithelium cells during infection by *Helicobacter pylori* and synovial rheumatoid arthritis [17–19]. Experiments using animal models also showed that IL21R-deficient mice had failures in antibody production and reduced cytotoxic T lymphocyte response [14]. IL21R is also expressed by mature adipocytes and was shown to regulate lipolysis [20]. Moreover, IL21R and IL21 play a role in the proliferation, migration, and invasion of breast cancer cells [21]. However, the role of IL21R in human ADSCs is poorly understood. Therefore, our study is aimed at examining protein localization in ADSCs and explore the role of IL21R in these cells by silencing and overexpressing it. Furthermore, we seek to evaluate possible alterations in cell proliferation, mitochondrial activity, and adipogenesis.

## 2. Methods

### 2.1. Isolation, Culture, and Differentiation of Adipose-Derived Stem Cells

In collaboration with the core for cell technology (PUCPR), we isolated, cultured, and characterized adipose-derived stem cells (ADSCs) from adipose tissue obtained by liposuction of subcutaneous abdominal (for a detailed description, see [22]). General information about gender, age, height, and weight of donors is provided in Supplementary Table S1. Tissue donors' consent was obtained under the guidelines for research involving human subjects and with the approval of the Ethics Committee of Fundação Oswaldo Cruz, Brazil (CAAE: 48374715.8.0000.5248). All tests were performed with cell cultures at passages 3–5.

### 2.2. Immunofluorescence

ADSCs were plated on sterile glass coverslips in 24-well plates for confocal microscopy (Leica SP5 confocal microscope) visualization and 96-well plates for analysis by the Operetta CLS High-Content Imaging System (PerkinElmer®, Waltham, Massachusetts, USA). Cells were washed three times with phosphate-buffered saline (PBS) at 37°C for 5 minutes and fixed in Methanol (Merck) -20°C for 10 minutes. Cells were incubated with PBS/0.5% Triton at room temperature for 30 minutes for permeabilization. We used 1% PBS/BSA as a blocking solution for 1 hour. The primary antibodies anti-IL21R 1 : 25 (10533-I-AP, ProteinTech), anti-ATP5B 1 : 25 (SC-55597, Santa Cruz Biotechnology), and anti-ALDH4A1 1 : 50 (sc-398911, Santa Cruz Biotechnology) diluted in PBS/BSA 1% were incubated overnight at 4°C. We performed incubation with secondary antibodies Alexa Fluor 488 (Sigma) or Alexa Fluor 546 (Sigma) 1 : 400 for one hour at room temperature and protected from light. We used DAPI 1 *μ*g/*μ*L diluted in PBS for five minutes for nuclei staining.

### 2.3. Overexpression of IL21R

Overexpression was performed using the GenEZ ORF clone of IL21R (NM_021798) constructed with pcDNA3.1+/C-(K)-DYK vector (GenScript). Transfections were performed in 6-well plates using Lipofectamine 3000 reagent (Invitrogen) to deliver plasmid DNA into ADSCs following manufacturer's protocol. The final concentrations were five *μ*g of DNA, 3.75 *μ*L of Lipofectamine 3000 Reagent, and 10 *μ*L of P3000 in 2 mL of media. As a negative control for the clones, a truncated IL21R clone was used. To obtain the truncated plasmid, the restriction digest manufacturer's protocol for the restriction enzyme HindIII (New England Biolabs) was followed. HindIII digestion generates a truncated version with a deletion at nucleotides 1 to 457 in IL21R CDS, removing the amino acid sequence of the extracellular portion containing the N-glycosylation and disulfide bond sites.

### 2.4. Short Interference RNA Assays

The three unique 27mer siRNA duplexes for IL21R (Origene-SR323990) were diluted to 20 *μ*M following manufacturer's instructions. Trilencer-27 Universal Scrambled Negative Control siRNA Duplex (SR30004) was used as a negative control for silencing experiments, also referred to as “scramble.” Transfections were performed in 6-well plates using 10 nM IL21R siRNA or scramble siRNA and 5 *μ*L Lipofectamine 3000 reagent (Invitrogen) following manufacturer's protocol. After 24 hours posttransfection, mRNA and protein were measured by qPCR and Western blotting.

### 2.5. Reverse Transcription-Quantitative Polymerase Chain Reaction (RT-qPCR)

Total RNA extraction was performed using the RNeasy mini kit (Qiagen). About 1 *μ*g of RNA was used to synthesize cDNA using an Improm-II Kit (Promega) according to manufacturer's instructions. The relative expression of specific genes was evaluated by RT-qPCR using LightCycler® 96 Instrument (Roche) and SYBR Select Master Mix (Life Sciences). The samples were incubated at 95°C for 30 seconds, followed by 35 cycles of 60°C for 30 seconds and 72°C for 30 seconds. The melting process then consisted of incubation at 72°C for 10 minutes. The sequences of primers used in this study were F5′ tcctggaaatgtggaacctcc3′ and R5′ tggcctcgtccttcagct3′ for *IL21R*; F5′ gaggactgagcatcgagca3′ and R5′ catgtgatctgacaccctgaa3′ for *STAT3*; *F*5′ ggcgatgctggcgctgagtac3′ and R5′ tggttcacacccatgacga3′ for *GAPDH* and F5′ taccacgtcatctcctttgatggct3′ and R5′ gtgcggctgcttccataa3′ for *POLR2A*. The RT-qPCR experiments were performed in triplicate. The *Cq* results for each gene were normalized based on GAPDH or POLR2A expression, and the relative expression of each gene was calculated.

### 2.6. Western Blotting

The cells were harvested and centrifuged at 700 x *g* for 10 min at 4°C. The supernatant was removed, and the pellet was treated with denaturing buffer (160 mM Tris-HCl pH 6.8, 4% SDS, 10% b-mercaptoethanol, 24% glycerol 24%, and 0.02% bromophenol blue). Lysates were separated by 13% sodium dodecyl sulfate-polyacrylamide gel electrophoresis (SDS-PAGE) and transferred onto a nitrocellulose membrane. The rabbit anti-IL21R antibody (1 : 200; Proteintech), rabbit anti-GAPDH antibody (1 : 1000; Cell signaling), and IRDye® 800CW Goat anti-Rabbit IgG Secondary Antibody (1 : 10000) were used as secondary antibodies. The infrared signal was captured with LI-COR Odyssey equipment. ImageJ software (https://imagej.nih.gov/ij/) was used for the quantitative analyses, and the gel images were quantified based on the linear signal ranges.

### 2.7. Proliferation Assay

Cell proliferation was measured using the Click-iT EdU Flow Cytometry Assay Kit. After treatment with siRNA or plasmid transfection, the cells maintained at 90% confluence into 6-well plates were incubated with 10 *μ*M EdU for 24 h in DMEM supplemented with 10% SBF. As a negative staining control, cells from the same population that had not been treated with EdU were used. After 24 hours of incubation, the cells were harvested and prepared for flow cytometry analysis according to manufacturer's protocol (Click-iT™ EdU Alexa Fluor™ 647 Flow Cytometry Assay Kit, Invitrogen™). Quantitative analyses of EdU-labeled cells were performed using a FACSCanto II flow cytometer (BD Biosciences) and Flow-Jo analysis software (FlowJo™ version 10.6.3).

### 2.8. Mitochondrial Membrane Potential (ΔΨ) Evaluations

In order to evaluate mitochondrial membrane potential (ΔΨ), cells were incubated with 50 *μ*M Rhodamine 123 (Life Technologies®), for 15 min at 4°C (protected from light), washed, and resuspended in PBS. Fluorescence intensity was measured by flow cytometry as previously described and analyzed with FlowJo software.

In a second approach to measure ΔΨ, 24 h after transfection (siRNA or overexpression), cells were incubated with the mitochondrial probe JC-10 (Sigma Aldrich). This dye forms aggregates with red fluorescence (*λ*ex = 540/*λ*em = 590) within cells with polarized mitochondria, whereas in cells with depolarized mitochondria membrane, the dye is monomeric and green-fluorescent (*λ*ex = 490/*λ*em = 525 nm). The ratio between red and green fluorescence was used to measure ΔΨ in cells at basal (Δ*F*) and uncoupling states (ΔFcccp). Briefly, cells at each experimental condition were first incubated with JC-10, and the Δ*F* ratio was measured. Cells were then treated with the uncoupling agent CCCP (carbonyl cyanide 3-chlorophenylhydrazone, 0.5 *μ*M) for 15 minutes at 37°C, and the *Δ*Fcccp was recorded. Fluorescence was quantified using the Operetta High-Content Imaging System. The Δ*F*/ΔFcccp ratio was calculated to obtain the relative ΔΨ at each experimental condition.

### 2.9. Adipogenic Differentiation and Quantification

For adipogenic differentiation, ADSCs were seeded in a 6- or 24-well plate (Jet Biofil) and were treated for different days (1, 3, 7, 10, or 14 days) with a basal medium containing the differentiation inducers for adipogenesis (Lonza) following manufacturer's instructions. As a control, cells were maintained with basal medium without adipogenesis inducers. To determine the grade of adipogenic differentiation and lipid accumulation, cytoplasmic triglycerides in the cells were detected by staining with Nile Red (N3013, Sigma). Briefly, the cells were washed in PBS, then fixed with 4% paraformaldehyde for 10 min. The cells were then washed twice with PBS, stained for 30 min with Nile Red (1 : 1000), washed again with PBS, and stained for 10 min with DAPI (1 mg/mL) (molecular probes). The stained cells were examined under the Operetta CLS High-Content Imaging System and examined using Harmony 4.8 software (PerkinElmer). A total of 16 photos were acquired per well at 20x magnification in biological triplicate. To quantify the nuclei, the images were acquired on the DAPI channel (355–385 nm excitation and 430–500 nm emission). Nuclei with circularity > 0.9 were considered in the analysis to exclude cellular debris. Images were acquired on the Alexa 488 channel (460–490 nm excitation and 500–550 nm emission) for determining the percentage of Nile Red-stained cells, intensity, and the number of lipid vesicles.

### 2.10. Statistical Analysis

Statistical analysis was performed with GraphPad Prism version 7.0. Experimental differences between scramble and siIL21R or pcDNA and IL21R overexpression ADSC were determined by Student's unpaired *t*-test analysis for mRNA expression analysis, protein quantification, proliferation, mitochondrial, and differentiation assays. Experimental data of qRT-PCR during differentiation were evaluated by two-way ANOVA with multiple comparisons. The data indicate the mean ± SD, and *p* < 0.05 was considered statistically significant.

## 3. Results and Discussion

### 3.1. IL21R Exhibits Noncanonical Subcellular Localizations in ADSCs

**Figure 1:**
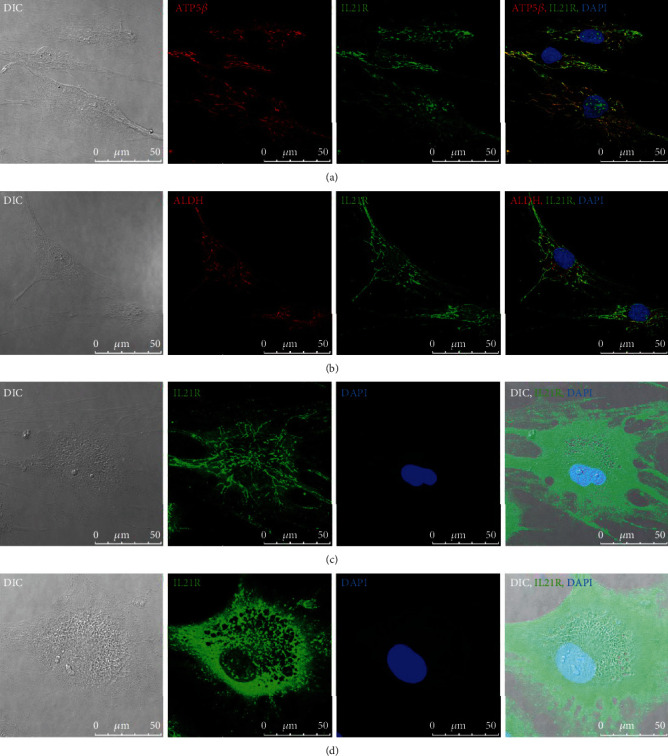
Intracellular localization of IL21R in ADSCs and induced to adipogenesis. Immunofluorescence labeling of undifferentiated ADSCs using *α*-IL21R, *α*-ATP5B, and *α*-ALDH4A1 in a confocal microscope (a, b). ADSCs were incubated with the primary antibody *α*-IL21R and the secondary antibody conjugated to Alexa488. ADSCs were incubated with the primary antibody *α*-ATP5B (Atp5b ATP synthase located in the mitochondria) or ALDH4A1 and the secondary antibody conjugated to Alexa 546. ADSCs were incubated with DAPI for nuclear staining. (a, b) Images were then merged and showed that *α*-IL21R is colocalized with *α*-ATP5B and ALDH4A1, indicating that the protein is located in the mitochondria and within cell nuclei. (c, d) Localization of IL21R in premature adipocyte (c) and in adipocyte containing more lipid vesicles (d). Scale bar: 50 *μ*m. Representative images of the donor TAL36 (a–d).

Although the presence of *IL21R* mRNA was previously demonstrated in human ADSCs [5, 10], the protein expression and intracellular localization of IL21 receptors remain unknown. Therefore, we performed immunostaining using anti-IL21R antibodies and analyzed them with confocal microscopy in order to precisely determine the localization of IL21 receptors in ADSCs (Figures 1(a) and 1(b)). The observed subcellular localization of IL21R has a mitochondrial pattern. To confirm these observations, we performed a colocalization experiment using an antibody specific for the beta subunit of the enzyme ATP synthase (ATP5B) and Aldehyde Dehydrogenase 4 Family Member A1 (ALDH4A1) (Figures 1(a) and 1(b)and Figure supplementary S1). The results confirmed the colocalization of the proteins IL21R with ATP5B and ALDH4A1 in the mitochondria of ADSCs (Figures 1(a) and 1(b)). Interestingly, we also identified the noncanonical presence of IL21R in the nucleus of ADSCs (Figures 1(a) and 1(b)). These results represent the first confirmation of the mitochondrial and nuclear location of IL21R in ADSCs. We evaluated the localization of IL21R after adipogenesis and found that despite the presence of lipid vesicles, IL21R maintained the same pattern of cell localization (Figures 1(c) and 1(d)).

IL21R and its ligand were first described 20 years ago as a class I cytokine receptor located in the cell membrane of lymphoid tissues [11]. To the best of our knowledge, this is the first report to demonstrate the colocalization of IL21R with mitochondrial proteins (ATP5 and ALDH4A1) in ADSCs using immunofluorescence assays, suggesting a possible role for IL21R in mitochondria. Noncanonical subcellular localizations have already been described for other receptors in different cell compartments. As an example, the canonical paradigm of G protein-coupled signaling implies that heterotrimeric G proteins function in conjunction with their cognate receptors located at the plasma membrane [23]. However, heterotrimeric G proteins may also have distinct functions in other cellular compartments [24]. For instance, G*α*12, one of the four families of *α* subunits of heterotrimeric G proteins, regulates mitochondrial motility, morphology, and membrane permeability [24]. IL21R may likewise have a noncanonical role in the mitochondria of hADSCs. Although there are no reports directly relating IL21R to mitochondria, the receptor and its ligand have been shown to participate in the regulation of metabolic reprogramming and T-cell differentiation. Specifically, it was shown that after T-cell activation, IL21 retains the oxidative phosphorylation-based metabolism typical of naïve T cells, unlike IL2, which induced aerobic glycolysis [25, 26]. ADSCs also undergo a metabolic reorganization during differentiation. After a short period of differentiation induction, ADSCs can change to an energetic and oxidative metabolic profile [27].

Interestingly, in our results, we also identified the presence of IL21R in the nucleus of ADSCs. In the last decade, a growing body of evidence has demonstrated the presence of various types of cell surface receptors, such as receptor tyrosine kinases (RTKs), peptide hormone receptors, and cytokine receptors, in the nuclei [28]. For example, EGFR physically interacts with STAT3 in the nucleus, leading to transcriptional activation of inducible nitric oxide synthase [29]. Wang and collaborators described a new role for IL21R in a nonimmune cell type. Using a mouse model for peripheral arterial disease, they showed that under hypoxia and serum starvation conditions, IL21R is upregulated in endothelial cells. Furthermore, IL-21 was found to promote signal transduction and activation of transcription 3 (STAT3) signaling, leading to an increased BCL-2/BAX ratio and increased endothelial cell survival and angiogenesis [30]. Also, IL21 binding to its receptor leads to the activation of multiple downstream signaling molecules, since STAT3 activated by IL-21 is vital for the differentiation of T follicular helper cells [31], while STAT3 activated by IL21 plays an essential role in B-cell differentiation and immunoglobulin class switching [32]. Further studies are needed to understand the role of IL21R in the nucleus and whether the receptor can physically interact with STAT3 in a manner analogous to EGFR. Considering these interesting results, we explored whether a loss or gain of function of IL21R might affect different cellular processes, such as the proliferation and mitochondrial activity in ADSCs.

### 3.2. Overexpression and Silencing of the IL21R Do Not Alter the Phenotype of Undifferentiated ADSCs

**Figure 2:**
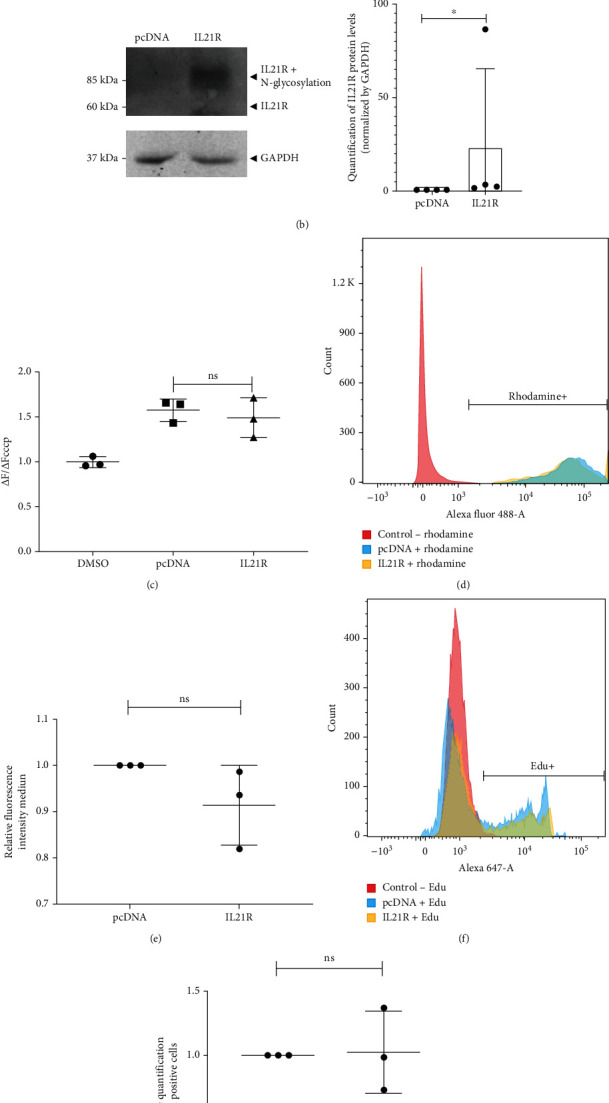
Overexpression of the *IL21R* gene in ADSCs. Cells were transfected with plasmid to overexpression of IL21R (IL21R), with a truncated plasmid (pcDNA) as a control. (a) Relative quantification of IL21R RNA normalized by GAPDH using qRT-PCR (*n* = 3) (TAL22, 27, and 32). (b) Western blot analysis to verify IL21R protein levels (*n* = 4) (TAL22, 27, 32, and 36). Protein levels of GAPDH were used as a control in the load, representative images of the donor TAL22. (c) Mitochondrial probe JC-10 measuring mitochondrial membrane potential (ΔΨ) in ADSCs with overexpressed IL21R (*n* = 3) (TAL23, 27, and 32). JC-10 fluorescence ratio at basal (Δ*F*) and uncoupling states (ΔFcccp) was used to measure ΔΨ. DMSO was used as a control. (d, e) Rhodamine 123 assay to measure ΔΨ after overexpression of IL21R (*n* = 3) (TAL23, 27, and 32). (d) Representative histogram with mitochondrial activity analysis. Cells without rhodamine (red curve) were used as a negative control. (e) Quantification of the relative fluorescence intensity means of rhodamine-stained cells. (f, g) Proliferation analysis using EdU incorporation in ADSCs with overexpressed IL21R (*n* = 3) (TAL22, 28, and 32). (f) Representative histogram with proliferation analysis by EdU incorporation. Cells without EdU (red curve) were used as a negative control. (g) Percentage of EdU-positive cells. Mean with SEM; Student's unpaired *t*-test analysis: ^∗^*p* < 0.05, ns: not significant.

In order to examine the role of IL21R in ADSCs, we overexpressed IL21R and evaluated its effects within the cell. ADSCs were transfected with pcDNA3.1 plasmid containing the *IL21R* gene or a truncated IL21R clone as a negative control. In order to verify the level of *IL21R* mRNA, we performed qRT-PCR 24 hours after transfection (Figure 2(a)), and the amount of IL21R protein was quantified by Western blot (Figure 2(b) and Figure Supplementary S2). We found a statistically significant increase in IL21R RNA and protein levels compared to the control (Figures 2(a) and 2(b)). The IL21R protein weighs 60.13 kDa and has five N- and one C-glycosylation site [13], each modification adding about ~2.5 kDa. Therefore, IL21R glycosylated at its six sites weighs approximately 85 kDa. An increase in the putative glycosylated form of IL21R protein could be detected in IL21R-overexpressed ADSCs compared to the control (Figure 2(b)). It was not possible to visualize changes in IL21R localization after IL21R overexpression (Figure Supplementary S3).

Given our colocalization findings, we evaluated the effects of IL21R overexpression on the mitochondrial membrane potential (ΔΨ) using Rhodamine 123 and JC-10 assays. These showed no significant alterations when compared to control (Figures 2(c)–2(e) and Figure Supplementary S5). Since proliferation is an elementary process, especially for stem cells, we evaluated the proliferation rate after IL21R overexpression using the EdU incorporation assay (Figures 2(f) and 2(g)) and by marking Ki67, a cell proliferation marker expressed in all stages of the cell cycle, except in the G0 phase (Figure supplementary S4). Our results indicated no significant changes in proliferative activity during a 24-hour culture period.

**Figure 3:**
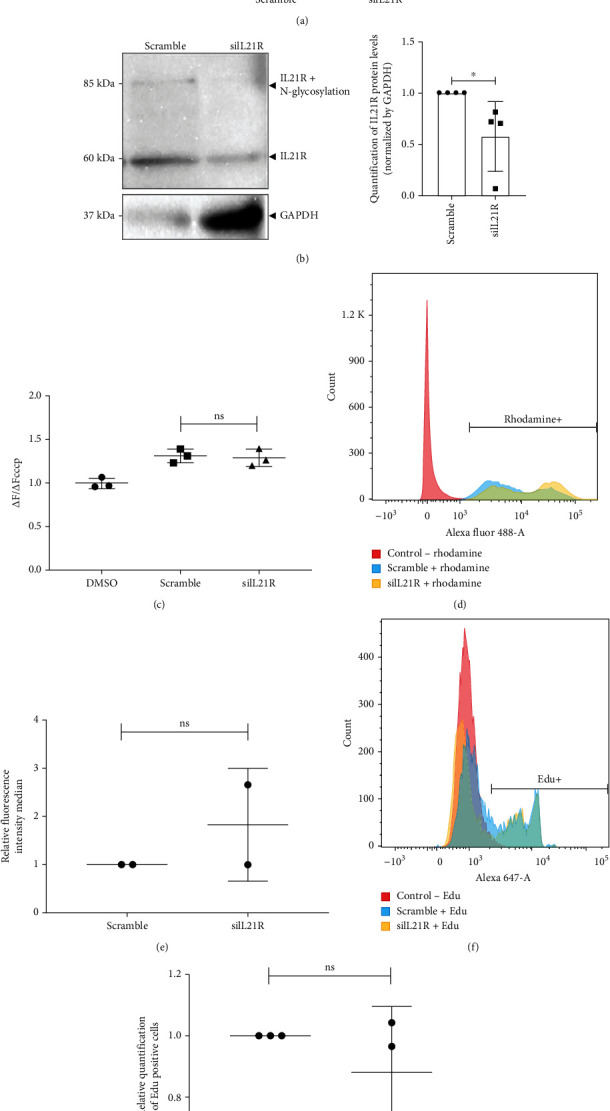
Silencing of the IL21R gene in ADSCs. Cells were transfected with siRNA of IL21R and a siRNA scramble as a control. (a) Relative quantification of *IL21R* RNA normalized by *GAPDH* using qRT-PCR after 24-hour transfection (*n* = 3) (TAL 22, 27, and 32). (b) Western blot analysis to verify IL21R protein levels (*n* = 4) (TAL22, 27, 32, and 36). Protein levels of GAPDH were used as a control in the load, representative images of the donor TAL22. (c) Mitochondrial probe JC-10 to measure mitochondrial membrane potential (ΔΨ) in ADSCs with gene silencing using IL21R siRNA and scramble as control (*n* = 3) (TAL23, 27, and 32). The JC-10 fluorescence ratio at basal (Δ*F*) and uncoupling states (ΔFcccp) was used to measure ΔΨ. (d, e) Rhodamine 123 assay to measure ΔΨ after silencing of IL21R (*n* = 3) (TAL23, 27, and 32). (d) Representative histogram with mitochondrial activity analysis. Cells without rhodamine (red curve) were used as the negative control. (e) Quantification of the relative fluorescence intensity means of rhodamine-stained cells. (f, g) Proliferation analysis using EdU incorporation in ADSCs after siRNA silencing (*n* = 3) (TAL22, 28, and 32). (f) Representative histogram with proliferation analysis using EdU incorporation. Cells without EdU (red curve) were used as a negative control. (g) Percentage of EdU-positive cells. Mean with SEM; Student's unpaired *t*-test analysis: ^∗^*p* < 0.05, ns: not significant.

As a second strategy to understand the role of IL21R in ADSCs, we performed IL21R gene silencing using the short interference RNA (siRNA) method. We then evaluated the phenotype of silenced cells for IL21R expression in mitochondrial activity and proliferation. After 24 hours of transfection, the expression of *IL21R* mRNA was significantly reduced relative to the negative control (scramble) (Figure 3(a)). We also observed a reduction in the IL21R protein level, which was measured by Western blot (Figure 3(b) and Figure Supplementary S3). It was not possible to visualize changes in IL21R localization after silencing IL21R (Figure Supplementary S3).

In an additional effort to understand the mitochondrial location of IL21R in ADSCs, we evaluated the possible effects of IL21R silencing on mitochondrial activity. Cells were incubated with the dye JC-10 and Rhodamine 123 to verify the relative mitochondrial membrane potential (ΔΨ). Our results suggest a nonsignificant difference in ΔΨ after IL21R gene silencing in comparison to the negative control in both mitochondrial probes (Figures 3(c)–3(e)). As a result, we suggest that the silencing of IL21R does not affect the mitochondrial activity of ADSCs.

To understand whether the reduction in IL21R expression affects the cell proliferation rate, we measured the incorporation of EdU and ki67 for siIL21R and the negative control (scramble). Our analysis demonstrates that IL21R silencing had no statistically significant effect on the proliferation rate of ADSCs over a 24-hour culture period (Figures 3(f)–3(g) and Figure Supplementary S4).

### 3.3. IL21R Is Regulated throughout Adipogenesis, and Its Silencing Impairs the Adipogenic Differentiation of ADSCs

We previously demonstrated, using RNA sequencing, that the level of IL21R mRNA associated with polysomes decreases during the initial steps of adipogenic differentiation (logFC = −1.72 and FDR = 8.22*E* − 04) [5]. Here, we quantified the total levels of IL21R mRNA at different times during adipogenesis (1, 3, 7, and 14 days) to validate this result. As expected, the expression of IL21R transcripts significantly decreases in the first 24 hours following induction compared to the control (no induced cells). Further on in the differentiation process (3, 7, and 14 days), no differences were found in IL21R expression between noninduced and induced cells.

**Figure 4:**
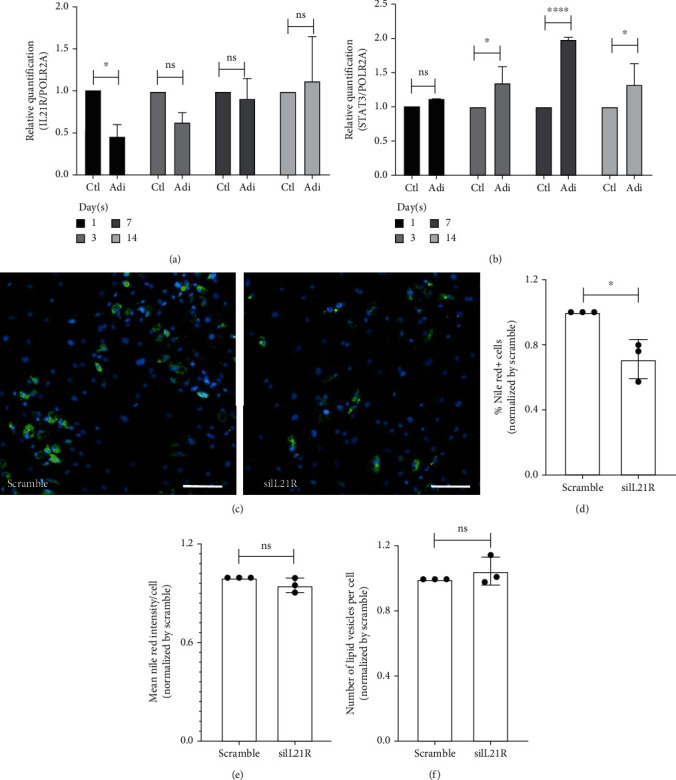
IL21R gene is modulated during adipogenesis, which is affected by gene silencing. (a, b) Relative quantification of mRNA *IL21R* and *STAT3* during adipogenesis in ADSCs, measured using qRT-PCR. The data were performed in biological and technical triplicate (TAL01, 09, and 32), normalized by *POLR2A*. Mean with SEM, two-way ANOVA with multiple comparisons: ^∗^*p* < 0.05, ^∗∗∗∗^*p* < 0.0001, ns: not significant. (c–f) siRNA-treated cells were induced to adipogenesis for ten days. The cells were then incubated with Nile Red (lipophilic fluorescent dye) and analyzed using the Operetta High-Content Imaging System (TAL23, 27, and 32). (c) Representative fluorescence images of ADSCs silencing IL21R and scramble after ten days of adipogenesis and stained with Nile Red (green staining) and DAPI (blue staining), representative images of the donor TAL23. Scale bar: 200 *μ*m. (d, f) Nile Red fluorescence quantification of ADSCs silenced for IL21R and induced to adipogenic differentiation for ten days. (d) Percentage of Nile Red positive cells, (e) mean fluorescence intensity per cell, and (f) the number of lipid vesicles per cell. Mean with SEM; Student's unpaired *t*-test analysis: ^∗^*p* < 0.05, ns: not significant.

The interaction of IL21 with its receptor triggers signaling transduction into cells through the JAK-STAT signaling pathway, which primarily involves the JAK1, JAK3, STAT1, and STAT3 proteins [15, 16]. Considering the critical role of the IL21/IL21R complex in the STAT3 signaling pathway, we highlight the fact that STAT3 is highly expressed in adipocytes and is a well-known participant in adipogenesis [33, 34]. We quantified the mRNA expression of STAT3 throughout adipogenesis, and we verified that its expression increases significantly after three days of induction and maintains this pattern until day 14 (Figure 4(b)). Liu et al. (2020) have demonstrated that IL21 can promote osteogenic differentiation by activating JAK3/STAT3 pathway in human valvular interstitial cells [35]. Therefore, we questioned whether IL21R had any role in the differentiation process.

Since IL21R seems to be regulated at the initial stages of adipogenesis, we wonder whether the receptor could influence adipogenic differentiation. To answer this question, we evaluated the adipogenic capacity of IL21R-silenced ADSCs. After 24 hours of siRNA transfection, ADSCs were induced to adipogenic differentiation. On the 10th day of differentiation, cells were fixed and labeled with Nile Red (Figure 4(c) and Figure Supplementary S6). Stained lipid vesicles were quantified by the Operetta High-Content Imaging System. Our results demonstrate that IL21R silencing reduced the percentage of cells stained with Nile Red (Figure 4(d)) suggesting that silencing of IL21R can interfere in the onset of adipogenesis when cells are committed to differentiation. Interestingly, the mean fluorescence intensity per cell (Figure 4(e)), the number of lipid vesicles per cell, and the expression of mRNA PPAR*γ*, a key regulator of adipogenesis (Figure Supplementary S6D), were not different between the scramble control and silenced cells (Figure 4(f)). The percentage of positive Nile Red cells was lower in cells overexpressing IL21 alone and IL21+IL21R, while there was no difference in cells overexpressing IL21R (Figure Supplementary S6A-C). More studies will be needed to understand the participation of IL21R during adipogenesis, especially with the presence of the ligand.

The limitations of this study were the discrepancy of results between the donors of cells, as well as the silencing of IL21R, which showed an amount of IL21R that could mask the effect in the different processes evaluated. It is necessary to evaluate the mitochondrial or proliferative activity in knockout cells or cells with overexpression of IL21R associated with its ligand, IL21, to be sure of the role of IL21R in these processes. Furthermore, studies of cell fractionation and transmission electron microscopy of mitochondria are needed to understand the location of IL21R and its role in this organelle. Here, we identified the influence of IL21R on the number of cells differentiated into adipocytes and without alteration in the expression of PPAR*γ*. More studies evaluating other genes associated with adipogenesis and other analysis times are needed to understand how IL21R interferes in the cell differentiation process. The amount of IL21R mRNA decreases in polysomes at the beginning of adipogenesis [5]. Here, we confirmed that result and showed that silencing the IL21R gene impairs adipogenesis. A previous study found that 72 hours from adipogenesis induction is the minimum time necessary for commitment and efficient differentiation [5]. Accordingly, the impairment of IL21R expression in ADSCs at the early stages of adipogenesis could compromise its commitment to differentiation. Considering that IL21R and its ligand are relevant to the JAK/STAT signaling pathway [36] and that STATs 1, 3, 5A, and 5B are expressed in adipocytes and regulated during adipogenesis (see reviews by [33, 34]) is conceivable that IL21R could be involved in adipogenesis through the activation of STAT3. Additional studies are necessary to confirm this hypothesis.

Several studies have indicated the Hedgehog (Hh) signaling pathway as an adipogenesis inhibitor [37–39]. The activation of the Hh pathway decreases the expression of IL21R mRNA, and its inhibition increases the level of IL21R mRNA associated with the translational machinery [10]. Consistent with these findings, our results suggest that the silencing of IL21R negatively affects adipogenic differentiation.

## 4. Conclusions

**Figure 5:**
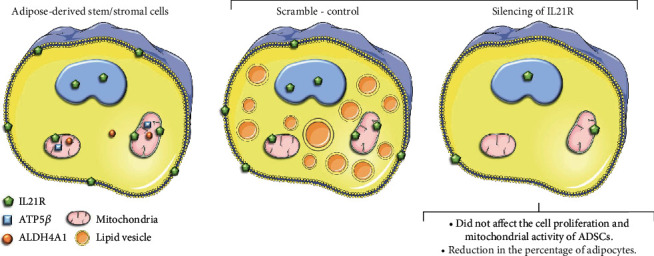
Schematic summary of findings on the role of IL21R in ADSCs and adipogenesis. The images were obtained from the Servier Medical Art (http://smart.servier.com).

To conclude, we found that noncanonical localization of IL21R occurs in the mitochondrial and nuclei of human ADSCs. Furthermore, IL21R silencing was able to reduce the adipogenic capacity of these cells (Figure 5). More studies are needed to understand the mechanism involved between IL21R and the adipogenic differentiation process.

## Data Availability

No data were used to support this study.

## Abbreviations

IL21R: Interleukin 21 receptor
ADSCs: Adipose-derived stem/stromal cells
ATP5B: A beta subunit of the enzyme ATP synthase
GAPDH: Glyceraldehyde-3-phosphate dehydrogenase
kDa: Kilodalton
SEM: Standard error of the mean.

## Supplementary Materials

Supplementary Materials Figure Supplementary S1: intracellular localization of IL21R in ADSCs. Immunofluorescence labeling of undifferentiated ADSCs using *α*-IL21R, *α*-ATP5B, and ALDH in a confocal microscope. (a, b) ADSCs (TAL27 and 32) were incubated with the primary antibody *α*-IL21R, and the secondary antibody conjugated to Alexa488, *α*-ATP5B (Atp5b ATP synthase located in the mitochondria), and the secondary antibody conjugated to Alexa 546 and *α*-ALDH4A1, and the secondary antibody conjugated to Alexa 546. ADSCs were incubated with DAPI for nuclear staining. Images were then merged and showed that *α*-IL21R is colocalized with *α*-ATP5B and ALDH4A1, indicating that the protein is located in the mitochondria and within cell nuclei. Scale bar: 50 *μ*m. Figure Supplementary S2: intracellular localization of IL21R in ADSCs after its silencing or overexpression. Immunofluorescence labeling of undifferentiated ADSCs using *α*-IL21R (green) and DAPI (blue), representative images of the donor TAL23. (a) Control ADSCs; (b) cells transfected with scramble; (c) cells transfected with siIL21R; (d) cells incubated without primary antibody. (e) Cells transfected with the control plasmid (pcDNA); (f) cells transfected with plasmid to IL21R overexpression; (g) cells transfected with plasmid to IL21 overexpression; and (h) cells transfected with plasmids to IL21R and IL21 overexpression. Total of nine photos were concatenated from each condition. Scale bar: 200 *μ*m. Figure Supplementary S3: detection of level IL21R protein in ADSC silenced with siIL21R (a) and IL21R overexpression (b) by using Western blot. Protein levels of GAPDH were used as a control in the load. *N* = 4. Figure Supplementary S4: Ki67+ cells in ADSCs with silencing (24 hs) or overexpression (48 hs) of IL21R are not affected by cell confluence. The proliferative profile of ADSCs submitted to IL21R silencing for 24 h or overexpression for 48 h were addressed by DAPI and Ki67 staining under standard immunostaining procedures (Lyra et al. 2021). The images were taken using the High-Content Image System Operetta CLS (Perkin Elmer). Twenty-five photos were acquired for each well; nuclei were acquired for the DAPI channel (excitation 355-285 nm, emission 430-500 nm); Ki67 staining was acquired for the alexa488 channel (excitation 460-490 nm, emission 500-550 nm). The analyses were performed with the harmony software 4.8 (Perkin Elmer), excluding cells at the edges and selecting nuclei with circularity greater than 0.9. Secondary antibody control (a) and Ki67 positive control (b) are shown. The number of nuclei labeled with DAPI and the number of nuclei with the intensity for Alexa488 greater than 7000 arbitrary units of intensity were evaluated, corresponding to cells positive for the Ki67 marker based on secondary antibody control (c) and Ki67-positive control (d) for the Alexa488 intensity shown on nuclei region. To evaluate if cell confluence would interfere with proliferative cell profile under silencing (e) or overexpression of IL21R (f), the Ki67 expression was evaluated on 40% or 90% of cell confluence, data were evaluated by two-way ANOVA, without significance. (*n* = 3 independent donors) (TAL27, 32, and 36). Scale bar corresponds to 100 *μ*m. Reference: Lyra et al. Bismuth-based nanoparticles impair adipogenic differentiation of human adipose-derived mesenchymal stem cells. Toxicology in vitro 77, 105248. ns: not significant. Figure Supplementary S5: (a) CCCP titration: ratio 546/488 in the uncoupling state of ADSC submitted to different CCCP concentrations (0.01-2.5 *μ*M), in the presence of JC-10. Experiment was performed in technical triplicates and data analyzed by one-way ANOVA and Tukey's multiple comparisons test. (b, c) Basal and uncoupling states for control and transfected cells: ratio 546/488 in the basal and uncoupled (0.5 *μ*M CCCP) states for silenced (b) and overexpressing (c) cells. Experiment was performed in biological triplicates (TAL 23, 27, and 32) and data analyzed by two-way ANOVA and Dunnett's multiple comparisons test. ^∗^*p* < 0.05, ^∗∗^*p* < 0.01, ns: not significant. Figure Supplementary S6: ADSCs were induced to adipogenesis for ten days. The cells were then incubated with Nile Red (lipophilic fluorescent dye) and analyzed using the Operetta High-Content Imaging System. (a, b) Representative fluorescence images of ADSCs overexpressing IL21R, IL21, IL21R+IL21, and pcDNA after ten days of adipogenesis and stained with Nile Red (green staining) and DAPI (blue staining). Scale bar: 200 *μ*m. Representative images of the donor TAL23. (c) Relative percentage of Nile Red-positive cells. (d) Expression levels of PPAR*γ* in control (siNC = Scramble) and silenced (siIL21R) cells induced or not (ctrl) to adipogenic differentiation during 10 days. Experiment was performed in biological triplicates, and data analyzed by one-way ANOVA and Tukey's multiple comparisons test. ^∗^*p* < 0.05, ns: not significant. Table supplementary S1: ADSCs donor information. ND: no data.
