# Supplementary material for: Interleukin 21 Receptor Affects Adipogenesis of Human Adipose-Derived Stem/Stromal Cells
Source: Stem Cells Int. 2022 Jan 10;2022:4930932. doi: 10.1155/2022/4930932 (PMC8763493; doi:10.1155/2022/4930932)
Supplement: Supplementary Materials — Figure Supplementary S1: intracellular localization of IL21R in ADSCs. Immunofluorescence labeling of undifferentiated ADSCs using α-IL21R, α-ATP5B, and ALDH in a confocal microscope. (a, b) ADSCs (TAL27 and 32) were incubated with the primary antibody α-IL21R, and the secondary antibody conjugated to Alexa488, α-ATP5B (Atp5b ATP synthase located in the mitochondria), and the secondary antibody conjugated to Alexa 546 and α-ALDH4A1, and the secondary antibody conjugated to Alexa 546. ADSCs were incubated with DAPI for nuclear staining. Images were then merged and showed that α-IL21R is colocalized with α-ATP5B and ALDH4A1, indicating that the protein is located in the mitochondria and within cell nuclei. Scale bar: 50 μm. Figure Supplementary S2: intracellular localization of IL21R in ADSCs after its silencing or overexpression. Immunofluorescence labeling of undifferentiated ADSCs using α-IL21R (green) and DAPI (blue), representative images of the donor TAL23. (a) Control ADSCs; (b) cells transfected with scramble; (c) cells transfected with siIL21R; (d) cells incubated without primary antibody. (e) Cells transfected with the control plasmid (pcDNA); (f) cells transfected with plasmid to IL21R overexpression; (g) cells transfected with plasmid to IL21 overexpression; and (h) cells transfected with plasmids to IL21R and IL21 overexpression. Total of nine photos were concatenated from each condition. Scale bar: 200 μm. Figure Supplementary S3: detection of level IL21R protein in ADSC silenced with siIL21R (a) and IL21R overexpression (b) by using Western blot. Protein levels of GAPDH were used as a control in the load. N = 4. Figure Supplementary S4: Ki67+ cells in ADSCs with silencing (24 hs) or overexpression (48 hs) of IL21R are not affected by cell confluence. The proliferative profile of ADSCs submitted to IL21R silencing for 24 h or overexpression for 48 h were addressed by DAPI and Ki67 staining under standard immunostaining procedures (Lyra et al. 2021). The [file 4930932.f1.pdf]

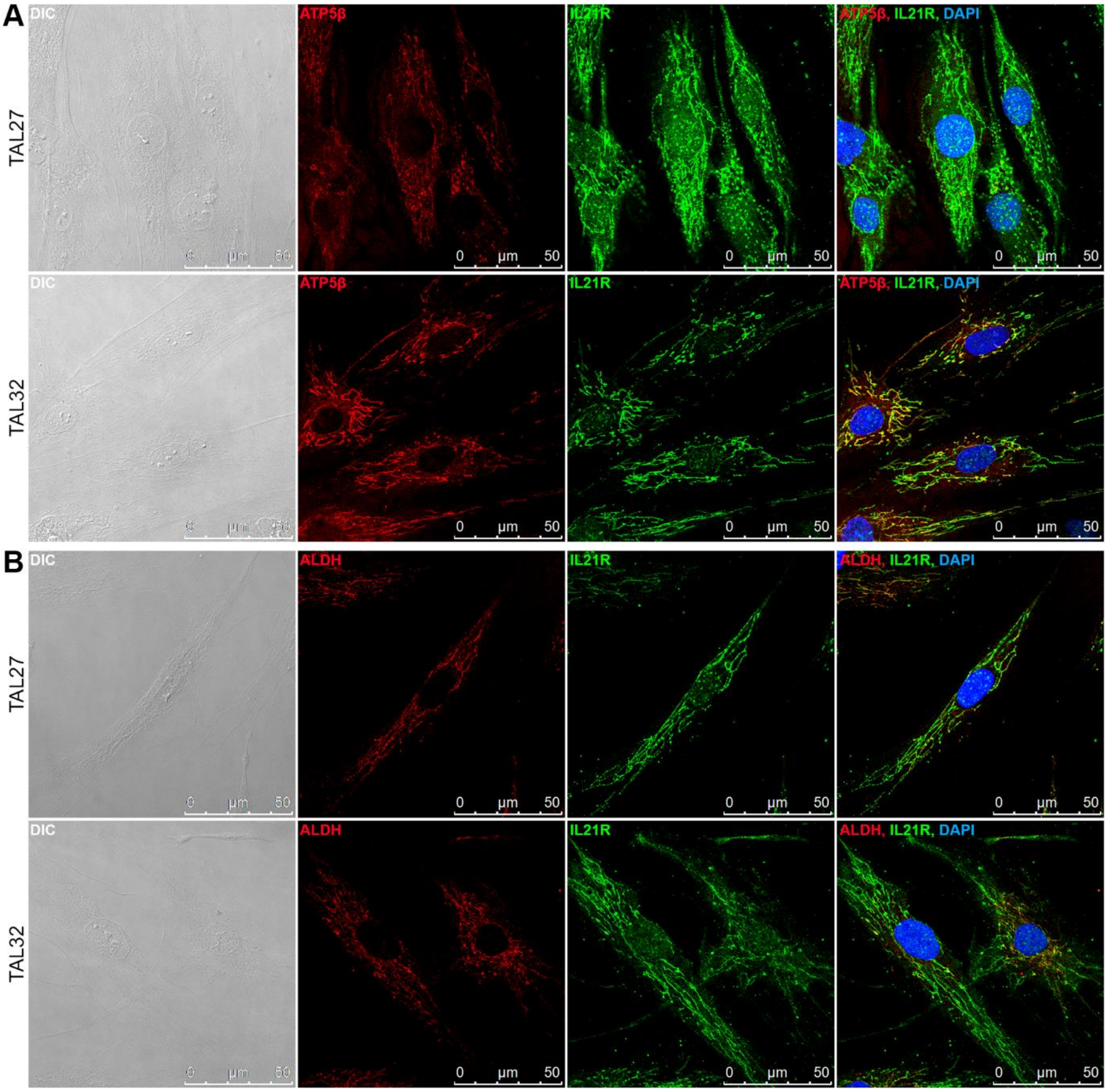

Figure Supplementary S1. Intracellular localization of IL21R in ADSCs. Immunofluorescence labeling of undifferentiated ADSCs using α-IL21R, α-ATP5B and ALDH in a confocal microscope (A-B) ADSCs (TAL27 and 32) were incubated with the primary antibody α-IL21R and the secondary antibody conjugated to Alexa488, α-ATP5B (Atp5b ATP synthase located in the mitochondria) and the secondary antibody conjugated to Alexa 546 and α-ALDH4A1 and the secondary antibody conjugated to Alexa 546. ADSCs were incubated with DAPI for nuclear staining. Images were then merged and showed that α-IL21R is colocalized with α-ATP5B and ALDH4A1, indicating that the protein is located in the mitochondria and within cell nuclei. Scale bar: 50 µm.

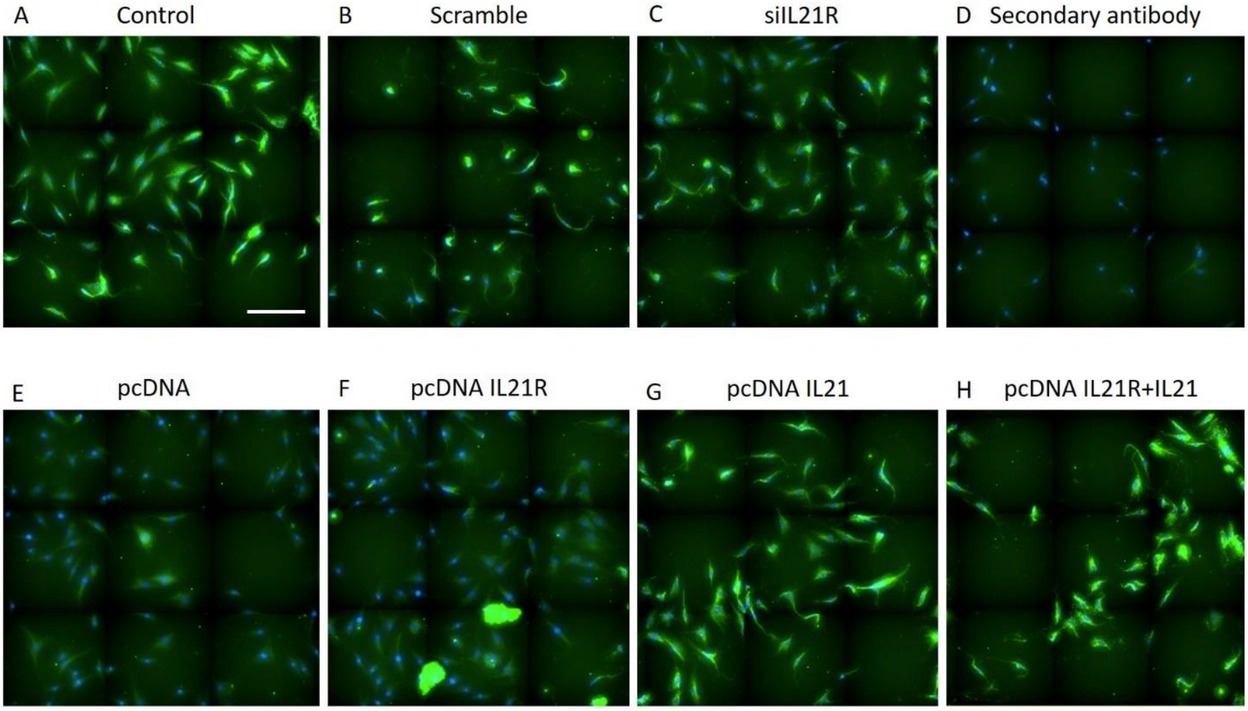

Figure Supplementary S2. Intracellular localization of IL21R in ADSCs after its silencing or overexpression. Immunofluorescence labeling of undifferentiated ADSCs using α-IL21R (green) and DAPI (blue), representative images of the donor TAL23. (A) Control ADSCs; (B) Cells transfected with Scramble; (C) Cells transfected with siIL21R; (D) Cells incubated without primary antibody. (E) Cells transfected with the control plasmid (pcDNA); (F) Cells transfected with plasmid to IL21R overexpression; (G) Cells transfected with plasmid to IL21 overexpression; and (H) Cells transfected with plasmids to IL21R and IL21 overexpression. Total of nine photos were concatenated from each condition. Scale bar: 200 µm.

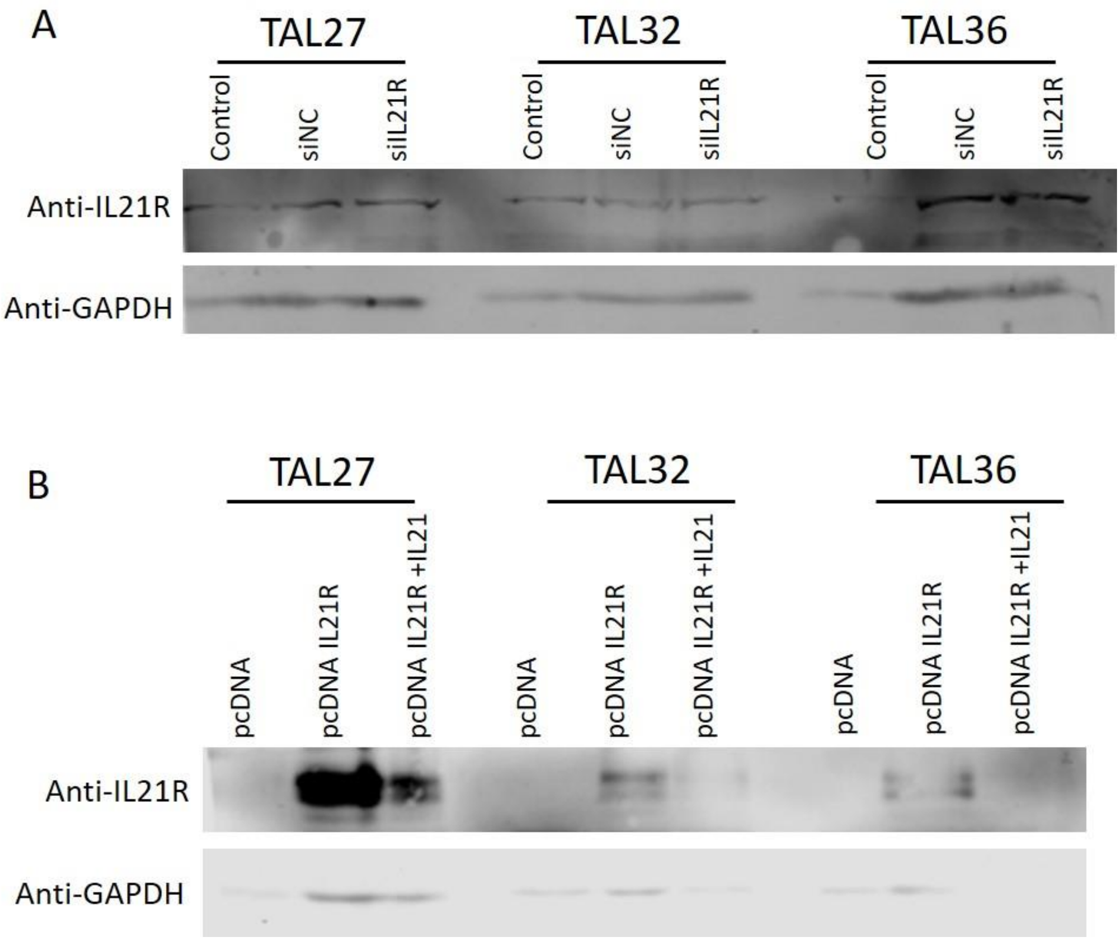

Figure Supplementary S3. Detection of level IL21R protein in ADSC silenced with siIL21R (A) and IL21R overexpression (B) by using Western blot. Protein levels of GAPDH were used as a control in the load. N=4.

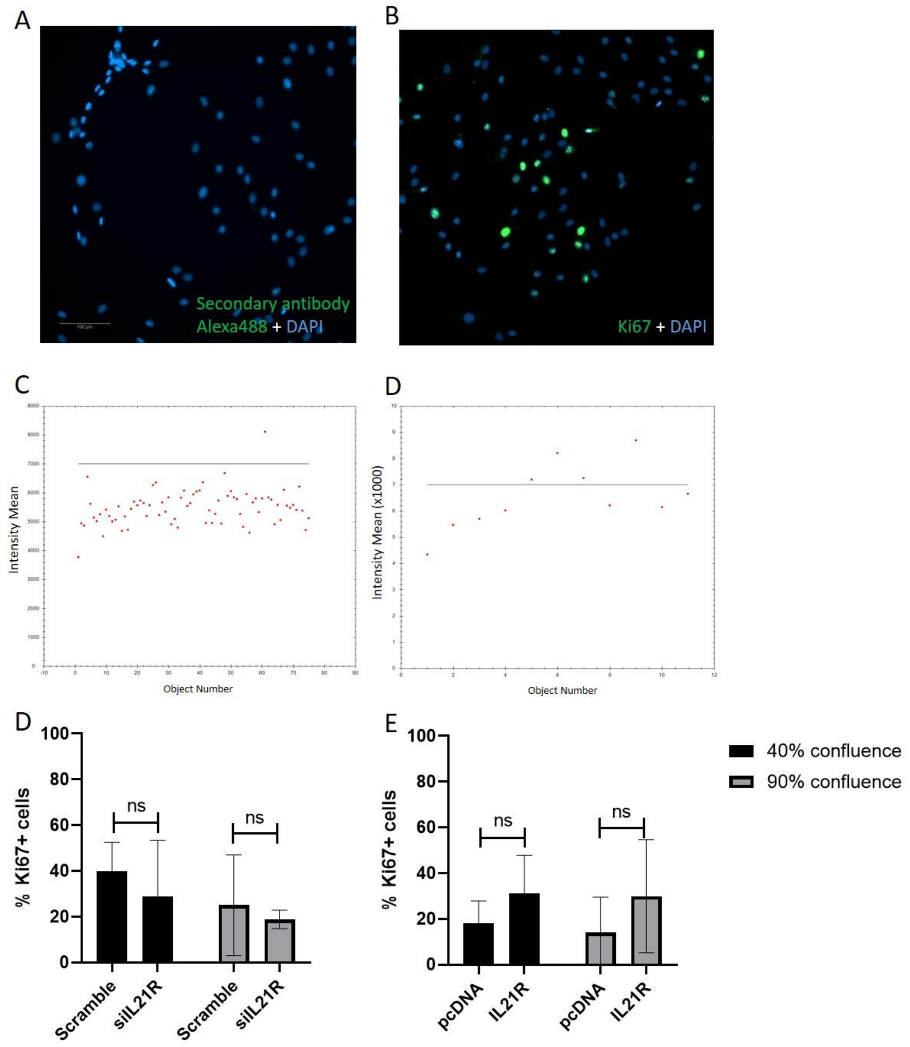

Figure Supplementary S4. Ki67+ cells in ADSCs with silencing (24hs) or overexpression (48hs) of IL21R are not affected by cell confluence. The proliferative profile of ADSCs submitted to IL21R silencing for 24h or overexpression for 48h were addressed by DAPI and Ki67 staining under standard immunostaining procedures (Lyra et al. 2021). The images were taken using the High Content Image System Operetta CLS (Perkin Elmer). Twenty-five photos were acquired for each well; nuclei were acquired for the DAPI channel (Excitation 355-285nm, emission 430-500nm), Ki67 staining was acquired for the alexa488 channel (Excitation 460-490nm, emission 500-550nm). The analyses were performed with the harmony software 4.8 (Perkin Elmer), excluding cells at the edges and selecting nuclei with circularity greater than 0.9. Secondary antibody control (A) and Ki67 positive control (B) are shown. The number of nuclei labeled with DAPI and the number of nuclei with the intensity for Alexa488 greater than 7000 arbitrary units of intensity were evaluated, corresponding to cells positive for the Ki67 marker based on Secondary antibody control (C) and Ki67 positive control (D) for the Alexa488 intensity shown on nuclei region. To evaluate if cell confluence would interfere with proliferative cell profile under silencing (E) or overexpession of IL21R (F), the Ki67 expression was evaluated on 40% or 90% of cell confluence, data were evaluated by Two-way ANOVA, without significance. (n=3 independent donors) (TAL27, 32 and 36). Scale bar corresponds to 100 µm. REFERENCE= Lyra et al. Bismuth-based nanoparticles impair adipogenic differentiation of human adipose-derived mesenchymal stem cells. Toxicology in vitro 77, 105248. ns= Not significant.

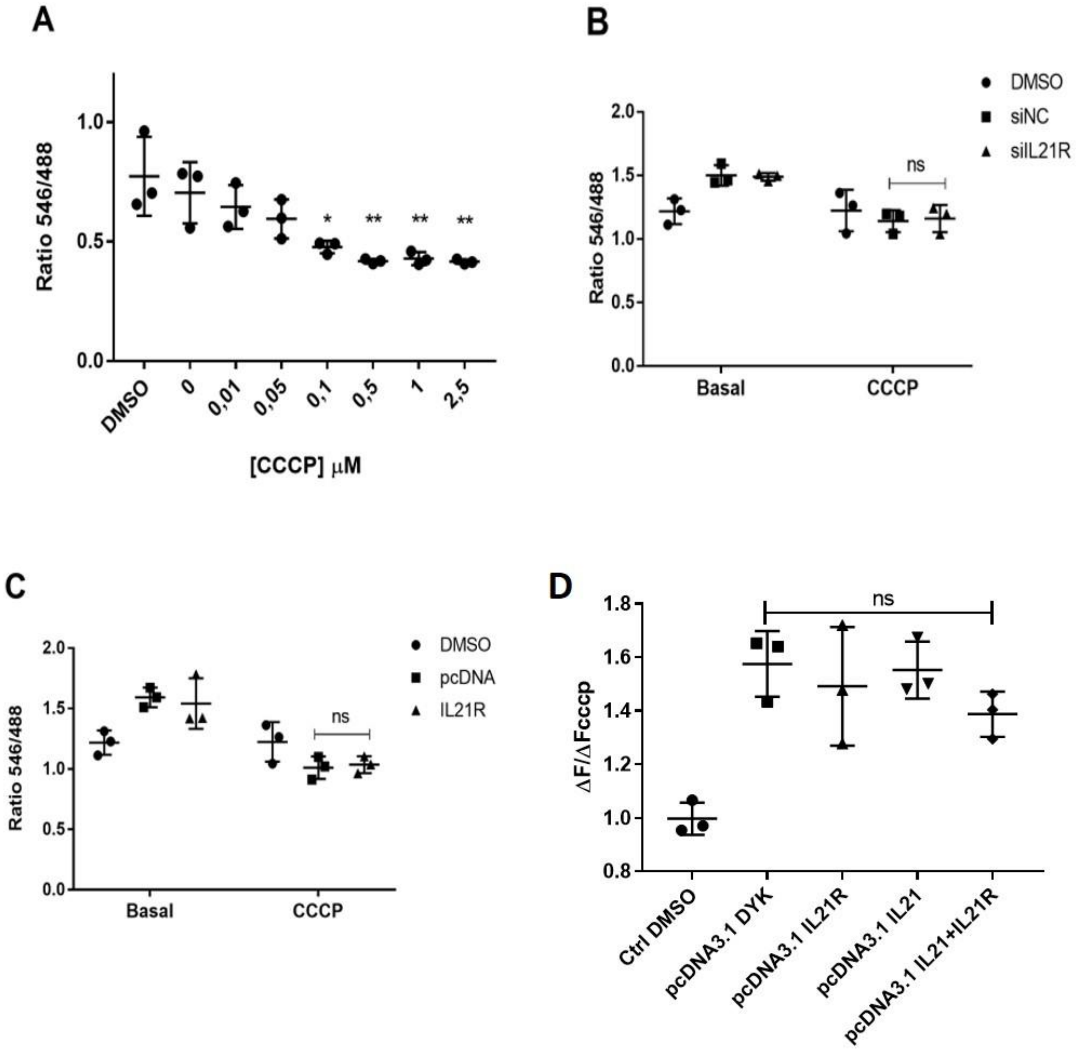

Figure Supplementary S5. (A) CCCP titration: ratio 546/488 in the uncoupling state of ADSC submitted to different CCCP concentrations (0.01-2.5 µM), in the presence of JC-10. Experiment was performed in technical triplicates and data analyzed by one-way ANOVA and Tukey's multiple comparisons test. (**B-C) Basal and uncoupling states for control and transfected cells:** ratio 546/488 in the basal and uncoupled (0.5 µM CCCP) states for silenced (B) and overexpressing (C) cells. Experiment was performed in biological triplicates (TAL 23, 27 and 32) and data analyzed by two-way ANOVA and Dunnett's multiple comparisons test. *$p<0.05$, **$p<0.01$, ns= Not significant.

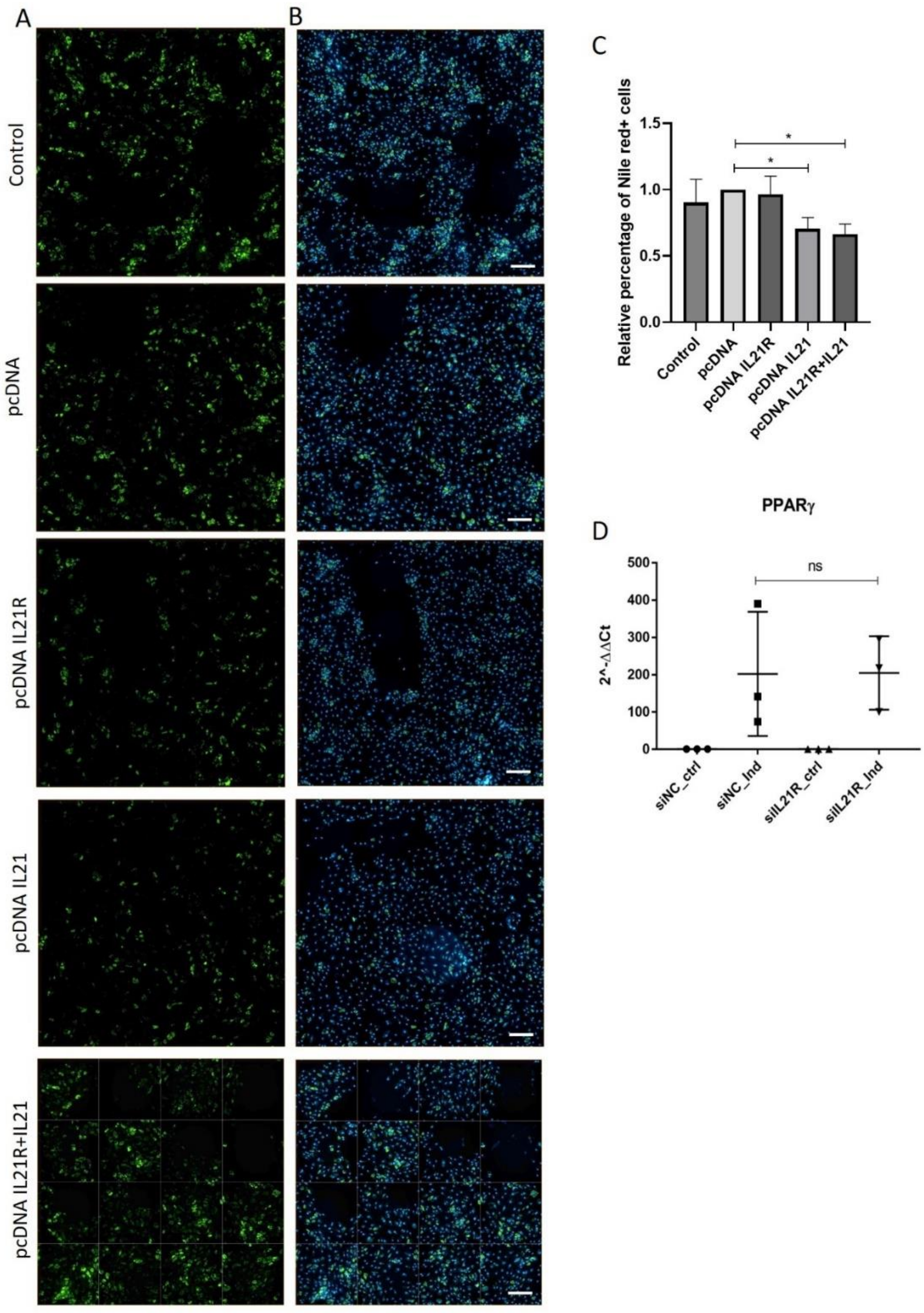

Figure Supplementary S6. ADSCs were induced to adipogenesis for ten days. The cells were then incubated with Nile Red (lipophilic fluorescent dye) and analyzed using the Operetta High-Content Imaging System. (A-B) Representative fluorescence images of ADSCs overexpressing IL21R, IL21, IL21R+IL21and pcDNA after ten days of adipogenesis and stained with Nile Red (green staining) and DAPI (blue staining). Scale bar: 200 µm. Representative images of the donor TAL23. (C) Relative percentage of Nile Red positive cells. (D) Expression levels of PPARγ in control (siNC = Scramble) and silenced (siIL21R) cells induced or not (ctrl) to adipogenic differentiation during 10 days. Experiment was performed in biological triplicates and data analyzed by one-way ANOVA and Tukey’s multiple comparisons test. *$p<0.05$, ns= Not significant.

Table supplementary S1. ADSCs donor information. ND = No data.

Donor	***Age***	***Gender***	***Height (m)***	***Weight (kg)***
TAL01	27	Female	1,60	70,9
TAL09	29	Female	1,69	66
TAL22	44	Female	1,63	72
TAL27	46	Female	1,66	74,5
TAL28	20	Female	1,74	75
TAL32	48	Female	1,75	90
TAL36	45	Female	ND	58
